# Athletes’ nutritional demands: a narrative review of nutritional requirements

**DOI:** 10.3389/fnut.2023.1331854

**Published:** 2024-01-18

**Authors:** Adam Amawi, Walaa AlKasasbeh, Manar Jaradat, Amani Almasri, Sondos Alobaidi, Aya Abu Hammad, Taqwa Bishtawi, Batoul Fataftah, Nataly Turk, Hassan Al Saoud, Amjad Jarrar, Hadeel Ghazzawi

**Affiliations:** ^1^Department of Exercise Science and Kinesiology, School of Sport Science, The University of Jordan, Amman, Jordan; ^2^Department of Physical and Health Education, Faculty of Educational Sciences, Al-Ahliyya Amman University, Amman, Jordan; ^3^Department of Nutrition and Food Technology, School of Agriculture, The University of Jordan, Amman, Jordan; ^4^Department of Family and Community Medicine, Faculty of Medicine, The University of Jordan, Amman, Jordan; ^5^Department of Nutrition and Health, College of Medicine and Health Sciences, United Arab Emirates University, Al Ain, United Arab Emirates; ^6^Oxford Brookes Center for Nutrition and Health, Faculty of Health and Life Sciences, Oxford Brookes University, Oxford, United Kingdom

**Keywords:** nutrition, athletes, carbohydrate, protein, micronutrients, fat, hydration, ergogenic aids

## Abstract

Nutrition serves as the cornerstone of an athlete’s life, exerting a profound impact on their performance and overall well-being. To unlock their full potential, athletes must adhere to a well-balanced diet tailored to their specific nutritional needs. This approach not only enables them to achieve optimal performance levels but also facilitates efficient recovery and reduces the risk of injuries. In addition to maintaining a balanced diet, many athletes also embrace the use of nutritional supplements to complement their dietary intake and support their training goals. These supplements cover a wide range of options, addressing nutrient deficiencies, enhancing recovery, promoting muscle synthesis, boosting energy levels, and optimizing performance in their respective sports or activities. The primary objective of this narrative review is to comprehensively explore the diverse nutritional requirements that athletes face to optimize their performance, recovery, and overall well-being. Through a thorough literature search across databases such as PubMed, Google Scholar, and Scopus, we aim to provide evidence-based recommendations and shed light on the optimal daily intakes of carbohydrates, protein, fats, micronutrients, hydration strategies, ergogenic aids, nutritional supplements, and nutrient timing. Furthermore, our aim is to dispel common misconceptions regarding sports nutrition, providing athletes with accurate information and empowering them in their nutritional choices.

## 1 Introduction

Achieving peak physical condition and optimizing athletic performance significantly hinge on the role of food (1). Optimal nutrition is instrumental in supporting physical activity, enhancing sports performance, and facilitating post-exercise recovery. Professional athletes are expected to comprehensively meet their nutritional requirements by consuming foods in appropriate quantity and quality (2).

A properly balanced diet generally provides adequate energy to sustain individuals with heightened energy needs due to physical activity, ensuring a maintained energy balance in most cases (3). However, meeting the energy needs of larger athletes with increased body weight and height, as well as athletes involved in demanding, high-volume training, can present a significant difficulty. Endurance athletes such as runners, swimmers, as well as those in sports like gymnastics, wrestling, and boxing that use dietary restriction for body composition adjustments, often face a negative energy balance (3, 4).

Sports nutrition refers to the nutrients found in sports-related foods that enhance physical capabilities. These nutrients form the foundation for meeting the metabolic requirements of athletes or bodybuilders engaged in regular physical exercise, contributing to the preservation of physical health and athletic performance (5).

The nutritional needs of athletes are primarily dictated by the demands of their activities and the goals they set to attain peak sports performance and overall health (2, 5). The age of athletes is critical, especially adolescents whose nutritional needs tend to be higher not only to cover the performance demands but due to growth demands also. Adhering to appropriate nutritional practices is crucial as it impacts nearly every bodily process, spanning from energy production to post-exercise recovery (6). Moreover, individual dietary choices may be influenced by factors such as knowledge, attitude, and the availability of nutrition-related information resources (7).

Dietitians need to consider various sport-specific factors when evaluating an athlete’s nutrition requirements and objectives. These factors include rules, arena size, competition timing, match frequency, and season length, which comprises macrocycles such as preseason, competition season, and off-season. Moreover, the physical attributes and position-specific responsibilities within the sport significantly impact the dietary requirements of athletes. For instance, rugby union forwards may require greater weight and strength compared to leaner and faster backs (8). Considering these sport-specific variables, variations in physique, and distinctions in positions, individualized dietary guidance becomes crucial for athletes participating in team sports.

Nutritional knowledge is a modifiable aspect that significantly shapes dietary behaviors (9). Athletes’ comprehension of sports nutrition can directly impact their food preferences, subsequently influencing their overall athletic performance (10–12). Therefore, athletes should seek reliable and certified sources for nutritional information, as this contributes to their understanding of nutrition (13, 14). A strategic approach to enhancing nutritional awareness among athletes involves the implementation of nutrition education programs (15, 16), including individual nutrition counseling, group workshops, or online educational materials (17–19).

Guidance that assists athletes in attaining adequate energy intake, achieving the right balance of macronutrients and micronutrients, and strategically timing their nutrition to enhance performance and recovery contributes to optimizing training and overall athletic performance (20). A previous review (21) revealed athletes in team sports frequently fail to meet the recommended dietary intake requirements (18, 19). Athletes who do not achieve sufficient energy intake and lack a well-balanced diet of macronutrients may encounter obstacles in both training adaptations and recovery processes (20, 22).

Inadequate energy intake can have adverse effects on an athlete’s performance, resulting in a reduction of fat-free mass, compromised immune function, decreased bone mineral density, heightened susceptibility to injuries, and an elevated occurrence of symptoms related to overtraining (20).

The field of sports nutrition has seen the publication of numerous new research papers, accompanied by the release of 17 fresh consensus statements and recommendation papers from authoritative organizations such as the IOC, ACSM, and ISSN (2, 20, 23–25). However, there is a recurrent observation of suboptimal adherence to sports nutrition guidelines among athletes (26–28).

Achieving optimal performance in sports demands meticulous attention to nutrition, where athletes must strategically examine the timing, amount, and nutritional value of their food consumption, coupled with ensuring sufficient fluid consumption. A synergistic blend of essential elements such as carbohydrates, proteins, fats, vitamins, and minerals plays a pivotal role, acting as the energy source, foundational components, and catalysts for success in the world of sports. This research paper endeavors to comprehensively examine existing literature on the nutritional needs of athletes, aiming to pinpoint and elucidate the specific nutritional demands essential for their peak performance.

Maintaining a healthy and well-balanced diet is of paramount importance for athletes and active individuals to optimize performance and overall well-being. The athlete nutrition guidelines under scrutiny in this manuscript play a pivotal role in guiding individuals to arrange their diet according to established recommendations. These guidelines not only serve the athletes themselves but also provide invaluable assistance to sports nutrition specialists in their work. The primary goal is to prevent disordered eating and eating disorders, which have become increasingly prevalent among the active population. As such, this manuscript seeks to comprehensively review current guidelines, offering a clear and updated overview. By doing so, it aims to empower athletes and active individuals to enhance their dietary practices, mitigating the risk of potential disordered eating.

## 2 Method

A narrative review was conducted over 6 months, spanning from November 2022 to April 2023. To ensure a comprehensive and relevant collection of studies, four prominent online databases–PubMed, Google Scholar, Scopus, and Web of Science–were meticulously explored. The search aimed to encompass a broad spectrum of articles pertaining to various facets of nutrition and its impact on athletic performance. To facilitate an exhaustive search, a combination of specific search terms and keywords was employed. These included “Intake of Carbohydrates for athletes,” “Intake of Protein for athletes,” “Intake of fat for athletes,” “Vitamins,” “Minerals,” “Hydration,” “Ergogenic Aids,” “Nutritional Supplements,” and “Nutrient Timing.” These keywords were selected to cover key areas of interest within the field of sports nutrition. The search strategy involved using different combinations of the aforementioned keywords to optimize the retrieval of pertinent literature. The review specifically included studies of an experimental nature, comprising randomized controlled trials, observational studies, case studies, and case reports conducted within groups of elite or semi-elite athletes. During the evaluation process, the authors scrutinized the articles to assess how well they addressed and aligned with the overall objectives of the literature review. Only those articles deemed highly relevant were included in the final selection.

## 3 Result

### 3.1 Energy (Kcal) and energy availability

In sports, maintaining peak performance relies heavily on precise nutritional practices and optimal energy intake. This is crucial not only for effective recovery and fatigue prevention but also to reduce the risk of injuries and illnesses (29).

Energy availability (EA) is a key concept, representing the dietary energy available for physiological functions after subtracting exercise energy expenditure. When there’s an imbalance, resulting in Low Energy Availability (LEA), athletes may face negative consequences across various physiological systems, including endocrine, cardiovascular, immune, metabolic, reproductive, and gastrointestinal (30).

Athletes persisting in a state of low energy availability may experience a range of issues, from disruptions in menstrual function to heightened focus on food, increased illness susceptibility, diminished mood, compromised performance, decreased libido, and hormonal imbalances (31).

While energy availability and energy balance seem similar, they differ fundamentally. Energy balance considers all components of energy expenditure, particularly relevant in changes induced by diet and exercise on body weight and composition. In contrast, energy availability focuses specifically on exercise energy expenditure, providing a nuanced perspective on the intricate relationship between nutrition and athletic performance (32).

### 3.2 Carbohydrate

A high proportion of carbohydrates in the diet can significantly enhance performance during endurance and intense training. This is achieved by increasing exogenous carbohydrate availability and storing carbohydrates, known as glycogen, in muscles and the liver (33). During training, there is a gradual depletion of endogenous carbohydrates due to energy expenditure; this dependence is contingent upon the level and length of the exercise. Rapid carbohydrate intake after exercise replenishes carbohydrate stores quickly and enhances the body’s training-induced adaptation processes. Among macronutrients, carbohydrates play a particularly crucial role in athletic performance since they can be metabolized aerobically and anaerobically (34, 35). Muscle glycogen and blood glucose are key energy sources for active muscles. Achieving optimal carbohydrate intake aids recovery and optimizes glycogen stores for subsequent training sessions. The recommended carbohydrate requirement varies with training volume and intensity. Emphasize the importance of incorporating foods exhibiting a glycemic index that extends across the spectrum from low to moderate into the meal plan, including complex carbohydrates (3). Nevertheless, during challenging and intense training sessions or when meeting high carbohydrate needs becomes difficult due to the substantial bulk and fiber content of complex carbohydrates, it is permissible to incorporate concentrated, nutrient-dense sources of carbohydrates. Additionally, low-risk supplements may be considered to meet daily requirements if necessary (3, 22).

The glycemic index functions as a tool to classify carbohydrate-containing foods according to their impact on blood glucose levels relative to glucose or white bread consumption (22). Its application in sports nutrition remains controversial, lacking clear recommendations for athletes. Studies indicate potential improvements in metabolism and substrate utilization during exercise when incorporating low glycemic index carbohydrate-containing foods in the pre-exercise meal (22). However, these findings do not consistently translate to enhanced exercise performance. Notably, the influence of glycemic index on the pre-event meal diminishes when carbohydrates are consumed during exercise (22). Therefore, choosing a low glycemic index meal before exercise might prove beneficial when restricted consuming carbohydrates during physical activity.

Endurance activities of moderate to high levels of intensity, along with resistance-based workouts, heavily depend on carbohydrates as a primary fuel source. Therefore, maintaining essential glycogen stores, around 80–100 g in the liver and 300–400 g in the muscles of the skeletal system, is crucial. Numerous studies confirm the limited nature of glycogen stores (36, 37), highlighting their pivotal role as a predominant fuel source for several hours during moderate engaging in aerobic exercise at a high level of intensity (38).

There is documented evidence that as glycogen stores decrease, it becomes harder to maintain a high pace of intense exercise (23). Consuming a snack or meal high in carbohydrates before exercise ensures optimal muscle glycogen reserves. On the other hand, low pre-exercise glycogen levels result in early fatigue, reduced training intensity, depleted muscle glycogen, impaired muscle contraction, glycogenolysis, and protein degradation (34, 39, 40).

A straightforward suggestion for maximizing internal glycogen reserves in high-performance athletes is to consume adequate carbohydrates based on the intensity and duration of their training. Generally, the suggested daily carbohydrate intake falls within the range of 5–12 g/kg of body weight. This is particularly relevant for athletes engaged during training sessions of moderate to high intensity, lasting over 12 h per week; it is recommended to target the upper limit of this range, precisely 8 to 10 g/kg of body weight daily (41). When substantial muscle damage is not present, this level of carbohydrate intake has demonstrated effectiveness in maximizing glycogen storage. Recommendations based on percentages (for instance, aiming for 60–70% of your total daily caloric intake to come from carbohydrates) have lost favor because they fail to accurately prescribe the required carbohydrate amounts for athletes with elevated caloric intake or those following restricted energy intake (33).

It is important to highlight that athletes frequently fall short of meeting the recommended levels of energy and carbs. Consequently (42), emphasizing approaches to restore glycogen reserves could be essential in preparing for optimal performance in the upcoming competition.

The synthesis of glycogen after exercise is closely tied to factors considering factors like the degree of glycogen reduction, the nature, duration, and intensity of the workout session (43). During the initial 6 h following exercise, there is an increased pace of muscle glycogen replenishment, and consuming sufficient carbohydrates within 24 h after the workout can lead to full restoration of glycogen stores (44).

Current studies suggest that having a recovery meal within 2 h after exercise, as opposed to not eating, proves effective in enhancing recovery (45). Despite optimal conditions, complete muscle glycogen resynthesis can extend up to 24 h, prompting studies to explore techniques for accelerating the pace at which muscle glycogen is restored (46). Customizing the approach based on the degree of glycogen depletion, an effective strategy for adequate glycogen resynthesis involves ingesting 1.0 to 1.5 g of carbohydrates per kg of body weight per hour right after exercise and maintaining this intake at 30-min intervals for up to 6 h following the workout (2, 47). On the contrary, in instances where carbohydrate (CHO) intake is postponed by 2 h, studies indicate a 45% reduction in glycogen resynthesis rates (48). The absorption of glucose in the intestines might serve as a constraint on glycogen resynthesis, especially notable when a significant quantity of carbohydrates is ingested in a single bolus after exercise (49). There is a proposition that carbohydrate (CHO) supplementation administered at a rate of about 1.0 g/kg/h, given at consistent intervals (15 to 60-min intervals) post-exercise, is more efficacious than a substantial single bolus in sustaining elevated blood glucose levels. This, consequently, facilitates increased muscle glycogen restoration (48).

Studies investigating glycogen resynthesis rates after varying carbohydrate consumption post-exercise yield inconsistent results (50). Van Loon et al. discovered that increasing post-exercise carbohydrate consumption–raising carbohydrate intake cyclists who consumed carbohydrates ranging from 0.8 to 1.2 g per kg per hour, in terms of body weight, at 30-min intervals exhibited increased rates of muscle glycogen synthesis (16.6 vs. 35.4 mmol/kg dry weight/h) following a glycogen depletion cycling trial (43). Current studies suggest that 2-h intervals may not be the most effective for promoting muscle glycogen resynthesis, especially given the rapid restoration observed within the initial 2 h post-exercise (43, 51). Additional studies have documented higher rates of glycogen resynthesis with more frequent supplement ingestion compared to a single large bolus (52).

Variations in study outcomes may arise from disparities in protocols; the effectiveness of post-exercise recovery meals is influenced by factors such as the timing and intervals of consumption, the subjects’ training status, and the type of carbohydrates used. Given these limitations and variations in research protocols, the current evidence indicates that an ingestion of carbohydrates ranging from 1.0 to 1.5 grams per kilogram per hour is recommended and is sufficient to achieve maximal glycogen resynthesis (2, 48, 50). Further research is essential, especially studies investigating the prolonged impacts of post-exercise nourishment and simulating a typical training program.

#### 3.2.1 Recommended daily intake of carbohydrates

To estimate athletes’ carbohydrate needs, a recommended guideline involves ingesting 3-12 g of carbohydrate consumption per kilogram of body weight each day, with the precise quantity contingent on the intensity and duration of their physical activity. Individual variations and the comfort of their digestive systems should also be considered. For approximate carbohydrate needs, please refer to Table 1 (35, 53).

**Table 1:** The approximate requirements for carbohydrates.

Exercise intensity	Minutes of activity per day	Recommended carbohydrate intake (g/kg)
Low	<60	3-5
Moderate	60	5-7
High	60-180	6-10
Very high	>180	8-12

The formula for calculating the daily intake of carbohydrates is as follows:


$$\begin{array}{l} \mathrm{Daily Intake of Carbohydrates}=\\ \;\mathrm{Weight in Kilograms}\;X\;\mathrm{Grams of Carbohydrates} \end{array}$$

### 3.3 Protein

Preserving and optimizing skeletal muscle mass are crucial goals for individuals with athletic aspirations, whether aiming for improved performance, increased muscularity, or accelerated recovery. Moreover, the importance of skeletal muscle mass extends beyond active individuals, providing direct clinical applications and benefits. This dynamic interplay–the balance between muscle protein synthesis and breakdown–is particularly crucial for aging adults, as skeletal muscle undergoes continuous regulation (54). The loss of muscle mass occurs when there is a negative balance, indicating a higher breakdown than synthesis, resulting in a net loss. On the flip side, the accumulation of muscle occurs when the rate of synthesis outpaces that of breakdown. The interaction between physical activity and dietary elements, particularly concerning the consumption of protein and indispensable amino acids, is pivotal in governing both the construction and degradation of muscle proteins (55). Recent findings indicate that alterations in the context of physical activity and nutritional intake have a more pronounced effect on muscle protein synthesis rates (56). Therefore, changes in muscle protein synthesis rates are deemed the principal determinants of variations in muscle mass over time as a response to both exercise and nutritional influences (57).

Amino acids are the structural constituents of proteins, providing the building blocks for all tissues. For athletes, the main purpose of consuming protein following vigorous exercise or competitions is the rebuilding and restoration involving both skeletal muscle and connective tissues (25). The amount, timing, and type of protein intake all affect the extent to which muscles remodel after training. It’s crucial to emphasize that protein is not the primary source of fuel for athletes (25).

Various factors, including the quantities of overall amino acids, indispensable amino acids, and BCAA concentrations, impact the anabolic effectiveness of a protein source. Additionally, factors such as protein digestibility, digestion rate, and absorption kinetics are taken into account. In the assessment of dietary protein quality, attention is often directed toward the indispensable amino acid composition offered by the protein source concerning human nutritional requirements. Additionally, its capacity for digestion, absorption, and assimilation by diverse tissues throughout the body is considered (58).

Two categories of protein determine whether they include essential amino acids: complete and incomplete protein sources. Animal protein is considered complete as it provides a comprehensive source of protein, containing all the required amino acids. In contrast, plant-based proteins are incomplete sources as they lack some essential amino acids (59).

As highlighted in a comprehensive review (60), biological values for prevalent plant sources typically fall within the range of 56–74, whereas various animal sources exhibit a spectrum spanning from 77 to 104 on theoretical scales ranging from 0 to 100 points. A similar discrepancy is noted regarding values of net protein utilization; plant sources typically fall within the range of 53–67, while animal sources tend to be in the range of 73–94 on a 100-point scale. The scores for Protein Digestibility Corrected Amino Acid stand out as one of the commonly utilized benchmarks for assessing protein quality (61) emerge as one of the most frequently employed metrics.

Casein, whey, and eggs achieve scores of 100 in their respective categories, serving as examples of animal protein sources, whereas red meat scores 92. In contrast, typical plant protein sources typically display Protein Digestibility Corrected Amino Acid stands values below 100, typically falling within the reported range of 45–75 (60). Except for soy protein, which has a score of 100, other plant sources generally fall below this threshold. Similarly, employing the DIAAS approach for evaluating protein quality reveals a consistent pattern: animal sources often exceed 100, in contrast, the majority of plant sources tend to be below this threshold. When analyzing the amino acid profiles of different plant-based isolates in comparison to standard proteins derived from animal sources and specimens from muscles in the human skeletal system, it becomes clear that several sources of protein derived from plants do not contain adequate quantities of specific amino acids, such as lysine and methionine levels (62). Moreover, they consistently exhibit reduced levels of indispensable and BCAA in contrast comparing animal protein sources to the amino acid composition present in human skeletal muscle. Furthermore, elements such as vary depending on factors such as the type of nutrient, individual characteristics, and the specific physiological context notably affect the nutritional quality of a protein. Concerning digestibility, compelling evidence suggests that numerous plant sources have a considerably lower digestibility rate (45–80%) compared to diverse animal proteins (>90%) (63). In essence, there is a general agreement that the transportation of amino acids to peripheral tissues from plant-based proteins is typically regarded as less efficient compared to that from animal proteins (64, 65). These distinctions are considered critical factors that influence the postprandial protein synthesis response observed in different tissues.

To promote muscle repair, remodeling, and improve post-exercise strength- and hypertrophy-related responses, it is crucial to ingest protein before, during, and after a workout (66). Consumption of protein during these periods has been associated with a favorable impact on Muscle Protein Synthesis (MPS) (67).

Prior to exercise, ingesting protein with meals within 3-4 h before the workout can assist in maintaining muscle growth and enhancing muscle recovery, especially when combined with resistance training (68). Combining amino acids with carbohydrates before exercise can lead to peak rates of MPS, although the effects of protein and amino acid feedings during this period on exercise performance are not firmly established. However, consuming carbohydrates combined with protein or essential amino acids during endurance and resistance training can have beneficial effects, including an improved anabolic hormonal status, reduced muscle damage, improved muscle cross-sectional area, and extended time to exhaustion (25).

It is important to note that protein has a limited capacity for the body to utilize as an energy source during activity, whereas carbohydrates are the primary fuel source. Therefore, rehydration and intake of simple carbohydrates (glucose) are most important for athletes during exercise (68).

To enhance myofibrillar protein synthesis after exercise and minimize amino acid degradation, a nutritional recommendation advises incorporating 0.31 g/kg of high-quality and quickly digestible protein, like whey protein. Those pursuing this objective should strive to integrate this amount per meal (69). A mixed meal consisting of carbohydrates and protein after exercise, with a carbohydrate-to-protein ratio of approximately 4 to 1, is recommended to initiate muscle glycogen synthesis (20). Note that exercise has a sustained anabolic impact for at least 24 h. However, the ideal timing for protein ingestion depends on individual tolerance as it may diminish over time after activity (70).

#### 3.3.1 The recommended daily intake of protein

Using the following recommendations in Table 2 may help the athlete to assess their need for protein and prevent excessive intake and keep them on track (74).

**Table 2:** Recommendation of protein intake based on the healthy athlete in different types of exercise and goals.

Type of training or exercise	The recommended amount of protein is typically expressed in g/kg of body weight
Preserving and building muscle mass (20)	1.4-2.0 g/kg of body weight
During caloric deficit periods (20)	2.3–3.1 g/kg fat-free mass
Endurance exercise (71, 72)	1.2-2.0 g/kg of body weight
Strength exercise (59)	1.6-2.8 g/kg of body weight
Gain (muscle) strength (73)	1.6-2.2 g/kg of body weight

### 3.4 Fat

To achieve optimal performance, athletes need to consume an appropriate amount of energy not only during exercise but also during recovery. Fat oxidation primarily depends on oxygen, while carbohydrate catabolism can occur with or without oxygen. It’s worth noting that the complete breakdown of glucose in the mitochondria, involving the presence of oxygen, yields more ATP (75). Consuming an adequate amount of fat is important, but high-fat or fat-loading diets are ineffective (76, 77). It is recommended to consume dietary fat between 20 and 35% of the total calorie intake for athletes, with saturated fat intake being less than 10% (76).

Athletes frequently turn to dietary supplements to boost metabolic capacity, delay the onset of fatigue, enhance muscle hypertrophy, and shorten recovery periods (76). Omega-3, a type of PUFA, acts as a structural component within cell membranes of phospholipids. Omega-3 plays a crucial role in the inflammatory response of the body (78). Among athletes, Omega-3 has been linked to the postponement of onset muscle soreness, enhancement of anaerobic endurance capacity, improvement in oxygen efficiency during aerobic exercise, support for skeletal muscle health, and mitigation of exercise-induced oxidative stress (79). It is recommended to take omega-3 after or with a high-fat meal for optimal absorption (79). The safe recommended dose for omega-3 is 450–900 mg/day, with a maximum recommended dose of up to 3 g per day (80).

Athletes have employed a dietary strategy of increasing the proportion of dietary fat, primarily aiming to enhance intramuscular triglyceride stores. The theory behind this approach suggests potential benefits for prolonged exercise performance while preserving glycogen stores (81). Endurance athletes, in particular, have considered and applied this strategy to improve their performance in prolonged exercises. Conversely, athletes focused on strength and power have given little thought to modifying fat intake in their training strategies.

Burke and her research team delivered compelling results, revealing that a five-day regimen of adhering to a high-fat diet, where over 65% of total calorie intake came from fat, while concurrently maintaining a low carbohydrate and a daily protein consumption of 2.5 g per kg of body weight, not only enhanced fat utilization but also enabled athletes to effectively partake in high-intensity and high-volume training sessions (82).

Moreover, sustained enhancements in fat utilization were observed even following the implementation of a regimen for carbohydrate loading aimed at replenishing muscle glycogen levels. This dietary strategy suggests that a sequence of high-fat intake followed by carbohydrate loading might establish a conducive environment, enabling skeletal muscle to oxidize more fat while maintaining sufficient muscle glycogen. Nevertheless, subsequent studies did not reveal improvements in exercise performance (83). Certainly, there was a noted decrease in the rates of muscle glycogen utilization throughout the exercise bout (84). Given the expectation that enhanced carbohydrate availability is likely to enhance power generation and exercise intensity, especially during the latter phases of prolonged exercise, these results were considered counterproductive.

While there has been extensive research on the effectiveness of high-fat diets, there is a general agreement that opting for a higher critical determinant, but rather, it depends on individual factors and the overall dietary context, an advisable approach to enhancing sports performance. In an extensive review of the literature, Johnson provided insights into how a high-fat diet affects performance in physical activities. These observations encompass (1): no definitive conclusions supporting the idea that decreasing intramuscular triglycerides adversely affects performance, a hypothesis intended to enhance performance (2); a diet rich in fats, comprising more than 46% of total calories derived from fat and less than 21% from carbohydrates, promotes fat oxidation through mechanistic adaptations. These adaptations encompass increased enzymes involved in the oxidation of fatty acids and improvements in both fatty acid transport and beta-oxidation; and (3) despite these mechanistic changes, improvements in exercise performance were not consistently observed, and in certain cases, a negative impact was evident (81).

While the suggestion of increasing dietary fat intake has been made for a positive impact on the utilization of substrates, the prevailing consensus discourages high-fat diets due to their adverse effects on performance. Instances of reduced carbohydrate utilization and gastrointestinal discomfort further reinforce the argument against such dietary approaches. Whether the negative outcomes arise from the elevated consumption of dietary fat or the probable simultaneous decrease within dietary carbohydrate, the adoption of diets rich in fats is not recommended.

### 3.5 Micronutrients

Micronutrients play a crucial role in sustaining life, encompassing vitamins and minerals that support well-being, development, and reproductive processes. These essential substances, required in small quantities, must be obtained through dietary intake as the human body cannot synthesize them (85).

Vitamins are categorized based on their solubility, with A, D, E, and K being fat-soluble, and B and C being water-soluble. Minerals, on the other hand, are non-organic compounds contributing to physiological operations (85). Daily physiological requirements classify minerals, with macrominerals requiring around 100 mg per day and trace elements approximately 20 mg per day for individuals’ health (85).

While a nutritionally balanced diet generally provides essential micronutrients in recommended doses for regular bodily functions, the appropriateness of these guidelines for athletes is a subject of debate. Athletes often resort to micronutrient supplementation, with close to half of them incorporating vitamin or mineral supplements in their regimen, according to a meta-analysis (86).

Research findings indicate that, similar to the impact of exercise intensity, duration, and character on macronutrient needs, these factors also influence athletes’ micronutrient requirements. In sports with elevated energy demands, there’s a likelihood of increased micronutrient needs, though precise quantification remains challenging (85).

When athletes have heightened overall energy requirements due to their training regimen, this increased need should reflect in both macro- and micronutrient intake. Meeting this demand through a well-rounded diet aligned with recommended dietary reference intakes for vitamins and minerals is generally achievable (87). However, certain scenarios, such as substantial losses through sweat and urine or specific dietary preferences, may lead to increased vitamin and mineral requirements (85).

In instances where athletes face challenges in meeting their micronutrient needs through diet alone, supplementation may be beneficial. Athletes might consider external supplements to enhance well-being and performance, especially in situations like altitude training, where iron supplementation may be necessary (88, 89). Specific sports may present unique concerns, such as lower concentrations of vitamin D for athletes in winter sports or indoor activities (90).

Athletes consuming a diet rich in energy from nutrient-dense foods typically do not require vitamin and mineral supplements. However, those who struggle to meet their micronutrient needs may benefit from supplementation, guided by a sports nutritionist (91, 92). It’s crucial to emphasize that while micronutrients are crucial for health, they do not exert ergogenic effects, and factors like low-energy diets, vegetarianism, illness, and injuries can contribute to micronutrient deficiencies (40, 93).

### 3.6 Hydration

It is crucial to replenish fluid loss during, before, and after exercise. Researchers have found that losing 2% of body fluids can affect performance and cognitive function. Thirst is often not an effective indicator of dehydration, as 1.5 L can be lost before thirst perception (94). Athletes are susceptible to losing 0.3 to 2.4 L per hour of sweat, which includes not only water but also salt, potassium, calcium, magnesium, and chloride. Consequently, fluid and electrolyte replacement should be incorporated into their recovery (93).

Fluid balance is fundamental for athletes, as hypohydration, which occurs when body water levels are lower than normal due to excessive sweating during exercise or diarrhea, can have life-threatening consequences and negatively impact performance (95). On the other hand, during endurance exercise, increased consumption of mostly sodium-poor or sodium-free liquids, such as water, can lead to hyponatremia (96). Excessive fluid consumption causes fluid retention in the body, resulting in dilutional hyponatremia (97). Athletes should be aware that the body can lose fluids in various ways through sweating as a natural result of prolonged exercise, urination, and other factors such as temperature and humidity (98).

The physical signs and symptoms of dehydration include dry and poor skin turgor, dark urine color, rapid weight loss, dry and sticky mouth, weakness, fatigue, headache, sunken eyes, muscle cramps, an increased rate of injuries, difficulty in recovery, and a racing heartbeat (99, 100). Additionally, over-hydration can manifest through physical signs and symptoms such as weight gain, swelling of the feet and hands (edema), nausea and vomiting, orthopnea (sensation of breathlessness during sleep), low blood sugar, weakness, seizures, fatigue, headache, and increased urination (99, 100).

Athletes are advised to initiate a pre-training regimen in a well-hydrated state, ingesting approximately 500 to 600 mL of water or a sports beverage 2 to 3 h before engaging in exercise. An additional intake of 200 to 300 mL of water or a sports drink is recommended 10 to 20 min before the onset of exercise (101). Throughout the training session, fluid replenishment should align with sweat and urine losses, endeavoring to sustain hydration levels while minimizing body weight reduction to less than 2%. Typically, this goal can be met by consuming 200 to 300 mL of fluid at intervals of 10 to 20 min. Post-activity, the focus of hydration should be on rectifying any fluid deficits incurred during the practice or competition (102).

Sports drinks are primarily used to rehydrate and replenish essential electrolytes and other important components for athletes, such as magnesium, sodium, calcium, potassium, glucose, and fluids lost during strenuous exercise, to enhance endurance and performance (103). On the other hand, the usage of sports drinks may differ based on the nature of the exercise. The best time to use a sports drink is during high-intensity and long-duration exercise lasting more than 1 h, on hot and humid days, during heavy or salty sweating, and for recovery after training (104).

The beverage hydration index model was introduced to evaluate the hydrating potential of a drink compared to plain water when individuals are at rest. This model operates on the assumption that a beverage inducing greater diuresis than water results in less retained available fluid in the total body water pool, reflected by a beverage hydration index below 1.0. Although a recent addition to beverage metrics, akin to the glycemic index for foods, the beverage hydration index has garnered replication by various research groups (105–107) since its inception (108). Significantly, population-specific factors like body mass and sex seem to have negligible effects, and the reproducibility of the hydration index model is reported to be robust (105). Consequently, the hydration index model has gained recognition as a reliable method for evaluating beverage hydration characteristics in well-controlled conditions, particularly when individuals are in a state of dehydration, as opposed to rehydration scenarios following exercise.

The addition of electrolytes to water seems to enhance fluid retention according to the beverage hydration index method (105, 109). Nevertheless, the minimum sodium level required to achieve this effect varies across studies, as some indicate that sports drinks with approximately 20 mmol sodium may not necessarily yield a significantly greater hydration index compared to the water control (106, 108). This aspect warrants further exploration, especially considering that sports drinks are commonly recommended for general public use as a suitable beverage for oral rehydration post-dehydration. Beverages with elevated sodium content (usually around 45 mmol), like Pedialyte, tend to exhibit a higher hydration index compared to water. However, whether Pedialyte has a hydration index superior to a sports drink remains uncertain in some studies (105, 107, 108). This aspect requires further investigation, especially considering that sports drinks are often recommended for general public use as a suitable beverage for oral rehydration after dehydration. Beverages with higher sodium content (typically around 45 mmol), such as Pedialyte, generally show a higher hydration index compared to water. However, whether Pedialyte has a hydration index superior to a sports drink remains uncertain in some studies (107, 108).

### 3.7 Ergogenic aids and nutritional supplements

In recent times, there has been a significant increase in attention devoted to nutritional supplements and ergogenic aids within the sports community. Nutritional supplements are concentrated reservoirs of nutrients or other compounds exhibiting nutritional or physiological qualities beyond what is naturally obtained through a regular diet (110). Ergogenic aids pertain to pharmaceutical substances employed to boost sports performance (111). Recognizing a profitable market, commercial brands cater to high-performance athletes (112), university students (113), and young amateur athletes (114) who enthusiastically supplement their diets with these products. The growing prevalence of athletes using nutritional supplements and ergogenic aids has raised concerns among health and sports authorities. A significant number of these supplements and aids have been found to be contaminated with harmful or banned substances (115). Such contamination could pose a risk to the health of athletes or lead to competition bans if the products contain prohibited doping substances (116). While the utilization of nutritional supplements and ergogenic aids is common among athletes ranging from recreational to elite levels, only a select few ergogenic aids (such as creatine, sodium bicarbonate, and caffeine) have been proven to enhance sports performance (117).

Dietary supplements play a crucial role in building muscle, boosting the immune system, and providing fuel to enhance training or athletic performance. Elite athletes often utilize performance-enhancing agents, with many considering supplements to be an essential component for sports success (118). A Canadian study revealed that 87% of elite male and female athletes used supplements regularly (112), while another study conducted in Canada found that 98% of young athletes between the ages of 11 and 25 used supplements on a regular or intermittent basis (119). In this section, we will discuss the effectiveness of the most widely used supplements for improving physical performance.

#### 3.7.1 Creatine

Creatine stands out as the most frequently used and scientifically backed ergogenic aid (120, 121). It holds a preferred status over other ergogenic aids due to its proven ability to increase power, enhance muscular strength, and promote an increase in fat-free mass, ultimately improving exercise and sports performance (121).

Recent studies highlight that creatine supplementation, with doses ranging from 0.3 g per kg per day for 3 to 5 days or 20 g per day for 5 to 7 days without interruption, results in a rapid increase in intramuscular creatine, providing ergogenic advantages (122). Additionally, creatine supplements have been shown to accelerate recovery from injury and muscle damage (122, 123). Notably, a study demonstrated that introducing a preload at 0.3 g/kg body weight and a post-maintenance protocol at 0.1 g/kg body weight after a vigorous eccentric resistance training session in young men led to reduced muscle damage and strength loss (124). Another study documented a substantial reduction in oxidative stress and an increase in athletic performance in male athletes who took creatine monohydrate (20 g/day) after 7 days (125). Previous research also suggests that creatine supplements can mitigate muscle damage resulting from prolonged, intense exercise sessions.

Studies on creatine supplements consistently show enhanced performance and increased strength in short-duration, maximal-intensity exercises, as evidenced by improvements in metrics such as single-repetition maximum, muscular strength, repetitions, muscular endurance, speed, and overall strength (126, 127). A meta-analysis examining the impact of creatine supplementation on upper and lower extremity performance revealed a noticeable increase in strength for both extremities (128). Notably, performance improvement was observed in individuals following a creatine supplementation program, particularly in conjunction with resistance training. This effect was particularly pronounced in individuals without a history of prior training, defined as those engaging in exercise less than 3 h per week.

Currently, the scientific literature strongly supports the utilization of creatine supplementation for boosting performance in short-duration, high-intensity resistance training, demonstrating a distinct influence on lean body mass. Nevertheless, it remains unclear whether these effects of creatine supplementation translate into enhanced athletic performance.

#### 3.7.2 Caffeine

Caffeine, a natural derivative stimulant, is associated with several proposed ergogenic effects. Known for its stimulating properties, caffeine not only improves performance but also increases the release of neurotransmitters, enhances intellectual ability, and boosts energy expenditure (129). Studies indicate that caffeine serves as a potent ergogenic aid for both aerobic and anaerobic training, particularly benefiting endurance activities like cycling and running (71).

Research suggests that consuming 2-5 mg/kg of caffeine before engaging in performance-based activities can significantly enhance sports performance (130). Another study demonstrated an approximate 3.2% improvement in athletic performance with similar doses of caffeine (131). It has been observed that moderate doses of caffeine, ranging from 3 to 6 mg/kg, are effective in improving sports performance, while high doses (≥ 9 mg/kg) do not show any additional benefits (80).

Several proposed mechanisms aim to explain the impact of caffeine supplements on athletic performance, focusing on their effects on endurance, muscle contraction, and perceived exertion (132, 133). The primary mechanism involves caffeine’s action within the central nervous system, where it competes with adenosine for its receptors, mitigating the adverse effects of adenosine on neurotransmission, arousal, and pain perception (134). Additionally, the analgesic effect of caffeine reduces the perception of pain and effort during exercise, potentially serving as an additional mechanism, especially in exercises inducing discomfort (132, 133). Consequently, reduced pain perception may contribute to sustained or increased motor unit firing rates, facilitating greater force production (133, 135).

The recommended dosage of 2-5 mg/kg aligns with existing literature, emphasizing the importance of optimal caffeine intake for maximizing benefits. The observation that high doses do not yield additional advantages suggests a dose–response relationship, emphasizing the need for moderation in caffeine consumption.

#### 3.7.3 Protein and amino acid supplements

Amino acid supplements enjoy widespread popularity and are commonly utilized by highly-trained athletes. Beyond the realm of sports, amino acids offer potential therapeutic benefits, such as promoting healing, enhancing the immune system, preventing muscle atrophy in both the elderly and malnourished individuals, and contributing to the treatment of kidney and liver diseases (136).

Critical for maintaining a positive nitrogen balance in the body, amino acid supplements, including branched-chain amino acids and protein powder, play an essential role (71). Approved by the FDA to counteract nitrogen loss, protein supplements are recognized as safe when used in accordance with good manufacturing or feeding practices (REF) (80).

#### 3.7.4 Whey protein

Following resistance training, incorporating whey supplements may contribute to enhanced muscle building. In a study, participants who consumed a 20 g whey supplement before and after resistance exercise exhibited greater gains in muscle mass and strength over a 10-week period compared to those who took a placebo (137). Another investigation revealed that athletes who consumed a whey supplement before and after a training session achieved the ability to perform more repetitions and lift heavier weights 24 and 48 h after the exercise, respectively (138).

It is crucial to emphasize that immediately after resistance training, the consumption of a high-quality protein source promotes muscle growth and aids in recovery (139). While whey supplements may be preferred over casein or soy in the immediate post-exercise period due to their faster absorption, there is no evidence suggesting that they result in greater muscle growth over a 24-h period (140). Additionally, whey protein has been associated with potential immune system benefits. Participants taking whey supplements experienced a smaller drop in glutathione levels, linked to lower immunity, after a 40-kilometer cycling time trial (141).

After intense exercise, additional protein is necessary to build new muscle proteins and repair damaged muscle cells (137). Current recommendations from scientists suggest athletes should consume between 1.3 and 1.8 g of protein per kilogram of body weight per day. The precise amount of protein required for muscle building has been a subject of debate, with strength and power athletes tending to consume at the higher end of this range (1.6–1.8 g) (103).

The study’s results support the idea that the timing and type of protein intake can influence post-exercise outcomes. While the faster absorption of whey is advantageous immediately after training, the overall 24-h impact on muscle growth appears comparable to other high-quality protein sources. Athletes can strategically incorporate whey protein into their post-exercise nutrition, recognizing its advantages while ensuring a balanced overall protein intake.

#### 3.7.5 Branched-chain amino acid

The best way to describe branched-chain amino acids (BCAAs) is as a combination of three out of the nine essential amino acids. Valine, leucine, and isoleucine, the three BCAAs, cannot be synthesized by the body on its own (142). These amino acids collectively constitute one-third of muscle proteins and play a pivotal role in the metabolism of skeletal muscle due to their distinctive properties (143). BCAAs facilitate the absorption of blood sugar by muscle fibers and influence insulin signaling (143). Notably, leucine is of particular importance among the three BCAAs, serving a crucial role in regulating muscle protein synthesis (MPS) and acting as a modulator even in the presence of hyperaminoacidemia (144).

Additionally, BCAA supplements operate through various mechanisms, including reducing soreness and preventing muscle tissue breakdown during resistance and intense training (145). They contribute to the reduction of central fatigue, promote muscle function recovery, and maximize the MPS response (145).

According to some studies, incorporating BCAAs before and after exercise may effectively prevent exercise-induced muscle damage and increase muscle protein synthesis (146). There is evidence suggesting that taking BCAA supplements before resistance training can also reduce delayed-onset muscle soreness and assist athletes in maintaining muscle mass during dieting (147).

However, it seems that endurance athletes may not significantly benefit from BCAA supplementation. A study conducted at Florida State University indicated that while taking a BCAA supplement before and during prolonged endurance exercise reduced muscle damage, similar effects were achieved by consuming a sports drink with carbohydrates (146). Another study involving long-distance runners found that, compared to a placebo, BCAA supplements taken 7 days prior to a marathon did not improve performance or reduce muscle damage (148). In essence, BCAAs do not appear to offer significant performance advantages during endurance exercises.

BCAAs, with a particular emphasis on leucine, play a crucial role in muscle protein synthesis and various aspects of muscle metabolism. The documented advantages of BCAA supplementation, such as reducing soreness, preventing muscle tissue breakdown, and enhancing recovery, align with their well-established role in supporting muscle function. While BCAAs demonstrate potential benefits in situations like resistance training and muscle preservation during dieting, their advantages may not be notably pronounced in the context of endurance exercises.

#### 3.7.6 Arginine

L-arginine, a non-essential amino acid naturally produced in the body, is commonly known by names such as arginine alpha-ketoglutarate (A-AKG) and arginine ketoisocaproate (A-KIC) (80). Numerous studies suggest that the performance of elite athletes during anaerobic exercise remains largely unaffected by arginine supplements (149). In a study focused on A-AKG supplements, athletes did not exhibit differences in nitric oxide (NO) levels, blood flow, or performance (150). However, a review of multiple studies indicated that arginine supplements might offer a modest benefit to novice athletes but not to more experienced athletes or female athletes (151). The recommended safe dose for arginine is up to 20 g per day (80).

While arginine is a naturally occurring amino acid, its supplementation seems to have a limited impact on elite athletes during anaerobic exercise, as suggested by several studies. The potential modest benefit for novice athletes, highlighted in a review, prompts further investigation into factors such as experience level and gender that may influence the effectiveness of arginine supplementation. The specified safe dose serves as a reference for individuals considering incorporating arginine into their nutritional regimen.

#### 3.7.7 Beta-alanine

Beta-alanine, a non-essential amino acid naturally produced in the body, increases muscle carnosine concentrations when taken as a supplement (152). Elevated muscle carnosine levels enhance buffering capacity, reducing lactic acid buildup during high-intensity exercise, which can improve performance in sprints and short distances by mitigating fatigue (153).

A systematic review of 19 randomized controlled studies has confirmed that beta-alanine supplements enhance performance in short, high-intensity activities (154). Analyzing 15 studies revealed an average performance improvement of 2.85%, translating to a 6-s enhancement over a 4-min event (155). Notably, runners who took beta-alanine supplements for 28 days in an Australian study significantly improved their 800-meter race times (156). In a United Kingdom study, amateur boxers receiving 6 g of beta-alanine per day for 4 weeks experienced a 20-fold increase in punch force and a four-fold increase in punch frequency (80). Many studies utilize daily doses of 3.2–6.4 g for six to 10 weeks (157). However, optimal results seem achievable with approximately 3 g (4 × 800 mg) per day for 6 weeks, followed by a maintenance dose of 1.2 g per day (156).

Beta-alanine supplementation has demonstrated efficacy in enhancing performance in short, high-intensity activities through increased muscle carnosine levels. The systematic review and specific studies provide robust evidence of its positive impact on various athletic parameters. The recommended dosage strategy underscores the significance of both the initial loading phase and the subsequent maintenance dose for optimal results. Athletes and individuals involved in high-intensity activities may consider beta-alanine supplementation as part of their performance enhancement strategy (158).

In summary, sports supplements lack systematic regulation, and there is no guarantee that they fulfill their claims or do not contain prohibited substances. Major sports organizations, including United Kingdom Sport, the US National Collegiate Athletic Association, and the International Olympic Committee (IOC), have policies advising against the use of sports supplements (159). It is recommended to prioritize a healthy diet and consult with your medical team or sports nutritionist before considering any supplements (160). Further research is essential to comprehend the combined effects of various sports supplement intake.

### 3.8 Nutrient timing

Nutrient timing involves strategically providing the appropriate macronutrients when the body is most primed to utilize them effectively (161). In the context of exercise, nutrient timing can be segmented into three distinct phases: the energy phase, the anabolic phase, and the adaptation phase.

The energy phase encompasses the period right before and during the exercise itself. Following exercise, the anabolic phase occurs immediately and typically spans about 60 to 90 min. This period, often referred to as the anabolic or metabolic window (161), highlights the heightened responsiveness of exercised muscles to nutrient intervention.

Subsequent to the anabolic phase, the adaptation phase unfolds. Consistently incorporating suitable supplements and meals during this period sustains an improved response to nutrient intervention for an extended duration. This fosters quicker recovery and facilitates training adaptation, enhancing overall exercise performance.

#### 3.8.1 Pre-exercise nutrition

The recommended timeframe for pre-exercise nutrition typically extends to the hour leading up to a training session, although some research has investigated the impact of consuming nutrients up to 4 h before engaging in physical activity (162). During this period, the primary objective of nutrient consumption is to ensure an adequate fuel reserve for the muscles, thereby enhancing performance during the exercise.

Explorations into pre-exercise nutrition trace back to the 1930s, when researchers began investigating physiological reactions during exercise in response to the intake of pre-exercise carbohydrates (CHO), such as glucose and fructose (163). As research progressed, studies delved into manipulating exercise performance through pre-exercise nutrition strategies.

An early study involving trained swimmers, employing different nutritional strategies, including supplemental cane sugar, did not reveal significant differences in performance (164). However, this study laid the groundwork for subsequent interventions and explorations in the field. Hargreaves et al. (165) found that consuming 75 g of CHO, either as glucose or fructose, 45 min before exercise did not confer advantages in time-to-exhaustion performance or glycogen utilization during 75% effort of maximal oxygen uptake in trained cyclists compared to a placebo. Conversely, a larger carbohydrate intake of 312 g, consumed 4 h prior to exercise, enhanced time trial performance at a 70% effort level VO2max after 100 min in individuals with recreational cycling training. However, these differences were not statistically significant when compared to meals with equivalent energy content comprising either 45 or 156 grams of carbohydrates (166).

In a separate study, significant improvements in a similar performance task were observed when recreationally trained individuals ingested either 1.1 or 2.2 g/kg of carbohydrates 1 h before exercise, in contrast to a placebo. Interestingly, no significant distinctions were noted between the two CHO doses (167) (33). Additionally, pre-exercise carbohydrate consumption has demonstrated an impact on substrate utilization during exercise, promoting glucose oxidation over free fatty acids even at low-to-moderate intensities (below 60% of maximal oxygen uptake, VO2max) (168) (34).

In summary, the collective influence of pre-exercise carbohydrate intake on endurance performance generally appears favorable, although findings across studies can be inconsistent. Interpretation of results should consider methodological aspects, including factors like the time elapsed since the last intense training session and existing muscle glycogen levels, which are interconnected and can influence the effectiveness of pre-exercise feeding.

The significance and performance-enhancing benefits of pre-exercise carbohydrates may be contingent on muscle glycogen content before feeding. This suggests that individuals with limited rest between training sessions may derive greater benefits compared to those with extended rest periods, provided they adequately consume carbohydrates. While much of the research has focused on aerobic exercises, there is growing evidence that activities involving high-intensity intervals, such as resistance exercise, may also experience advantages. These activities predominantly rely on glycolytic, fast-twitch muscle fibers, which generate force through rapid muscular contractions fueled by stored phosphagens and anaerobic glycolysis, leading to lactate production.

Even with around 40% total glycogen depletion after high-volume resistance exercise, it suggests that carbohydrate availability may not be a limiting factor unless glycogen stores are suboptimal. Importantly, substantial evidence indicates that pre-exercise supplementation with carbohydrates can mitigate glycogen reductions, even if it does not notably impact blood glucose levels (169).

#### 3.8.2 During exercise nutrition

Carbohydrate (CHO) intake during physical activity has been extensively studied since the 1960s (49). It plays a crucial role in mitigating the utilization of muscle and liver glycogen, especially in situations involving high exercise intensity, durations exceeding 60 min, or shorter, supramaximal exertions (170, 171). Insufficient CHO in these scenarios can lead to decreased exercise intensity due to a shortage of efficient fuel, diminished calcium release, and increased fatigue (172, 173). Inadequate carbohydrate intake during such activities may result in decreased exercise intensity due to a shortage of efficient fuel, diminished calcium release, and increased fatigue. Carbohydrate (CHO) oxidation rates from exogenous sources show an exponential increase in the initial 75–90 min of exercise, underlining the significance of commencing CHO ingestion from the start and sustaining it throughout the exercise session for the conservation of muscle and liver glycogen (174). Excessive CHO intake, on the other hand, may lead to gastrointestinal upset, potentially impeding performance goals. Diversifying CHO ingestion with different transporters can enhance CHO uptake and oxidation to approximately 1.5 g/min or 90 g/h (175). This varied CHO consumption not only improves CHO availability without causing gastrointestinal upset (176) but also carries the potential to enhance overall performance (177). For example, a study illustrated an 8% increase in power output during a time trial after 120 min of steady-state cycling when cyclists consumed beverages with a 2:1 ratio of glucose to fructose, as opposed to glucose alone (177). Importantly, fructose ingested at a rate of 1.2 g/min, when combined with an equal amount of glucose, leads to a higher CHO oxidation rate compared to lower quantities, in line with the commonly recommended 60 g/h. These results highlight the potential benefits of integrating varied carbohydrate (CHO) intake to enhance performance (171–174, 176, 177).

An alternative approach to optimizing carbohydrate (CHO) delivery, with the goal of minimizing gastrointestinal distress and potentially boosting performance, involves the simultaneous intake of protein and CHO. Recent findings from a review and meta-analysis indicated positive performance outcomes, especially in time trials or efforts to exhaustion, for groups consuming a combination of CHO and protein compared to CHO alone (178). The noted favorable effect persisted consistently, even with the utilization of non-isocaloric supplements. However, when ensuring that CHO and protein supplements were equivalent in CHO content and subsequent examination of the effects of isocaloric supplementation involving both CHO and protein or CHO alone on time to exhaustion, no notable differences were observed (178).

Although the simultaneous ingestion of protein and carbohydrates may not yield immediate performance improvements, there are indirect advantages. These encompass the capacity to boost caloric intake while reducing carbohydrate consumption to prevent gastrointestinal distress, enhancing amino acid bioavailability to reduce muscle protein breakdown, and improving amino acid availability for gluconeogenesis. Moreover, co-ingestion may play a role in postponing central nervous system fatigue (179).

The effectiveness of intra-exercise nutrition, especially the consumption of carbohydrates (CHO), is highly contingent on variables like pre-exercise feeding, glycogen status, and the type of exercise (178). For aerobic activities lasting more than 2 h, optimal results are achieved by consuming 90–144 g/h of CHO in a 2:1 glucose to fructose solution. This strategy maximizes the uptake and oxidation of CHO while simultaneously preserving muscle glycogen.

In competitive scenarios, where extended endurance events frequently conclude with a sprint to the finish line, relying significantly on anaerobic metabolism and the utilization of endogenous muscle glycogen, the prudent conservation of this fuel source throughout the entire bout becomes paramount.

#### 3.8.3 Post-exercise nutrition

After engaging in physical activity, individuals commonly experience a temporary surge. During this phase, there is an increase in fatigue, muscle soreness, and a decline in performance. In this stage, catabolic processes take precedence, leading to decreased insulin levels, restricted glycogen, and limited substrate availability. Cortisol and catecholamines collectively influence physiological processes in the body, heightening the pace at which muscle protein is being broken down (180).

The intake of carbohydrates and protein post-exercise offers the potential to raise glucose levels in the bloodstream, reduce cortisol levels, and improve substrate availability, enabling the transition from a catabolic state to a more anabolic condition (180). Additionally, activating muscle GLUT4 transporters, increasing glycogen synthase activity, and enhancing insulin sensitivity all contribute to improving how responsive skeletal muscles are to absorbing carbohydrates and amino acids (50, 181). Therefore, the post-exercise period offers a strategic opportunity for nutrient intake to aid in replenishing muscle glycogen, promoting protein synthesis, and reducing the degradation of muscle proteins (180, 181). Integrating the timing of nutrient intake after exercising into a training routine becomes essential for optimizing recovery rates and maximizing the benefits of training.

During moderate-to-high intensity exercise, muscle glycogen assumes a crucial role as the primary source of energy to sustain physical activity. In light of this situation, precise post-exercise nutrient timing becomes vital, emphasizing the primary goal of replenishing muscle glycogen to hasten the recovery process. After physical activity, there is a decrease in the heightened levels of post-exercise glucose transporters, which are crucial for the absorption of nutrients. This decline brings the transporter levels back to baseline within a two-hour period (182).

Aside from glycogen synthesis, the consumption of protein and essential amino acids following exercise plays a pivotal role in triggering muscle protein synthesis and aiding in the reconditioning of skeletal muscles (183). After exercising, there is a notable increase in muscle damage and protein degradation in the aftermath of exercise (161, 181). Moreover, when glycogen stores are depleted, the pace of protein breakdown increases, as amino acids could potentially undergo gluconeogenesis to be utilized in replenishing levels of glycogen (91). As a result, it is crucial to consume protein after exercise to mitigate the breakdown of proteins and assist in the repair of muscle damage (183).

When aiming to stimulate muscle protein synthesis, proteins that are rapidly digestible and of high quality, containing an adequate amount of essential amino acids, may be more effective than proteins with lower quantities of branched-chain amino acids or those that are slower to digest (69).

Comprehensive training and maintaining a sufficient daily protein intake are crucial for achieving strength and hypertrophy. However, beyond these foundational aspects, there are potential advantages to carefully considering the timing of protein consumption, especially immediately after exercising. The positive impacts on net protein balance and glycogen synthesis underscore the significant benefits of ingesting protein in the post-training period.

Fundamentally, critical factors contributing to optimal performance include not only the quality of the training but also the overall protein intake throughout the day. The strategic timing of protein consumption provides an additional layer of support to boost performance. Even if the resulting benefits are seemingly minor, this aspect becomes a pertinent factor, particularly for competitive athletes who are dedicated to optimizing their performance.

In summary, the significance of nutrient timing is a nuanced matter, and its relevance varies greatly depending on the context. Defined as the delivery of adequate macronutrients precisely when the body is ready to use them (184), nutrient timing represents a dietary approach where specific nutrients are ingested before training to enhance both short-term performance and long-term adaptations (23).

Early research delved into the effects of acute carbohydrate (CHO) consumption on exercise performance, focusing on glycogen depletion and use during moderate to high-intensity aerobic activity (185, 186). Subsequent studies broadened the scope to investigate how acute protein consumption (PRO) impacts endurance and resistance workout performance, as well as recovery and adaptation. Despite these inquiries, it’s noteworthy that the effects of nutrient timing on performance, recovery, and adaptation outcome variables have only been explored in a limited number of chronic interventional studies.

The time before exercise, typically within 4 h prior, involves specific dietary tactics. Glucose loading, for instance, aims to boost muscle glycogen reserves in the days preceding an endurance race, resulting in enhanced glycogen storage levels and improved performance in activities lasting over 90 min (167, 187).

Consuming 150–200 grams of carbs 4 h before exercise has shown to significantly increase muscle glycogen reserves, ultimately contributing to improved exercise performance (188–190). Additionally, it is advisable to include healthy fats in the diet 6-5 h before exercise. Conversely, to prevent gastrointestinal discomfort, it’s recommended to avoid fiber consumption 2-3 h before engaging in physical activity (67, 190, 191).

The energy phase during a workout is crucial as muscles require sufficient energy for contractions. According to timing science, consuming carbohydrates, proteins, specific amino acids, and vitamins 10 min before and during exercise is necessary for this objective. The benefits encompass a good supply of glycogen, a reduction in cortisol, and assistance in preparing muscle enzymes for faster recovery (192, 193).

Post-workout nutrition timing is widely regarded as the most crucial phase. Intensive resistance training sessions damage muscle fibers and deplete the body’s reserves of fuel, including glycogen and amino acids. Consuming the right balance of nutrients during this time initiates the healing process for injured tissue and replenishes energy stores. This occurs in a super-compensated manner, enhancing exercise performance and body composition. Researchers often refer to this limited timeframe following training as the “anabolic window of opportunity,” emphasizing its significance in maximizing muscle adaptations related to the training (194–196).

## 4 Conclusion

In conclusion, this narrative review offers targeted recommendations for addressing the nutritional needs of the active population, with a specific focus on preventing disordered eating. Given the unique challenges faced by athletes, it is imperative to tailor nutrition plans to individual requirements.

Individualization emerges as a cornerstone in preventing disordered eating among athletes. Recognizing diverse goals, body compositions, metabolic rates, and dietary preferences is essential. Tailoring nutrition plans to accommodate these individual factors can significantly contribute to optimizing performance while mitigating the risk of disordered eating (197).

Macronutrients, which include carbohydrates, proteins, and fats, play a critical role in athletic nutrition. Adequate carbohydrate intake is necessary to support energy production and replenish glycogen stores, thereby reducing the likelihood of restrictive eating behaviors (33). Proteins are indispensable for muscle repair and growth, emphasizing the importance of meeting increased protein needs without resorting to excessive dietary restrictions (198). Meanwhile, healthy fats contribute to sustained energy, hormone production, and overall health, promoting a balanced approach to nutrition.

In addition to macronutrients, micronutrients, encompassing vitamins and minerals, are paramount for energy metabolism and immune function. Promoting a diverse, nutrient-dense diet is crucial to ensuring athletes receive adequate micronutrients, thereby reducing the risk of nutritional deficiencies that might contribute to disordered eating (199).

Hydration emerges as a key factor in preventing disordered eating among the active population. Proper fluid balance is essential for physiological function, and athletes must be attuned to their individual fluid needs. Maintaining adequate hydration levels before, during, and after exercise is crucial, as dehydration can exacerbate disordered eating behaviors.

While acknowledging the interest in sports nutrition supplements, caution is advised. Athletes should prioritize meeting their nutritional needs through whole foods to minimize the risk of disordered eating patterns (200). Supplements should only be considered when dietary intake falls short or specific deficiencies are identified. Consultation with qualified professionals is essential to ensure safe and appropriate usage.

In summary, implementing these targeted nutritional recommendations can serve as a proactive tool in preventing disordered eating within the active population. By understanding and addressing the unique challenges faced by athletes, promoting individualization, and emphasizing a balanced and informed approach to nutrition, this review contributes to the overarching goal of investigating and preventing disordered eating in the active population.

This review delves into the most recent research findings on nutritional recommendations for athletes, offering readers a comprehensive overview of the current state of the field. By skillfully spotlighting significant patterns, accepted procedures, and novel studies, we provide an invaluable resource for both researchers and practitioners, enhancing the reader’s comprehension of the intricate connection between nutrition and athletic performance.

It is important to note that the authors’ judgment played a major role in the inclusion of studies. The absence of a systematic search and uniform inclusion criteria may lead to the inclusion of research with methodological flaws or the unintentional exclusion of pertinent studies. Additionally, subjectivity in the interpretation of results may have resulted in the overemphasis of some topics and the omission of others.

In summary, this review underscores the pivotal role of athlete nutrition guidelines in facilitating optimal dietary arrangements for individuals involved in sports and physical activity. By comprehensively reviewing existing guidelines, this manuscript aims to furnish a resource that benefits athletes directly and aids sports nutrition specialists in their vital work. The overarching objective is to cultivate an environment of informed dietary choices, contributing to the prevention of disordered eating and promoting the long-term health and performance of athletes and active individuals. As we navigate the intricacies of sports nutrition, the insights gleaned from this manuscript aspire to guide future research and interventions, ensuring a holistic approach to the well-being of individuals engaged in athletic pursuits.

## Author contributions

AdA: Conceptualization, Writing – review & editing. WA: Writing – review & editing. MJ: Writing – review & editing, Data curation, Software. AmA: Methodology, Validation, Writing – original draft. SA: Conceptualization, Data curation, Writing – original draft. AH: Validation, Writing – review & editing. TB: Investigation, Writing – original draft, Writing – review & editing. BA: Supervision, Writing – review & editing. NT: Methodology, Writing – review & editing. HS: Formal analysis, Writing – original draft. HG: Conceptualization, Investigation, Software, Supervision, Validation, Visualization, Writing – original draft, Writing – review & editing. AJ: ____.
